# Genome insight and description of antibiotic producing *Massilia antibiotica* sp. nov., isolated from oil-contaminated soil

**DOI:** 10.1038/s41598-021-86232-z

**Published:** 2021-03-23

**Authors:** Ram Hari Dahal, Dhiraj Kumar Chaudhary, Jaisoo Kim

**Affiliations:** 1 Department of Life Science, College of Natural Sciences, Kyonggi University, Suwon, Kyonggi-Do, 16227 Republic of Korea; 2 Department of Microbiology, School of Medicine, Kyungpook National University, Daegu, 41944 Republic of Korea; 3 Department of Environmental Engineering, Korea University Sejong Campus, Sejong City, 30019 Republic of Korea

**Keywords:** Biological techniques, Drug discovery, Microbiology

## Abstract

An ivory-coloured, motile, Gram-stain-negative bacterium, designated TW-1^T^ was isolated from oil-contaminated experimental soil in Kyonggi University. The phylogenetic analysis based on 16S rRNA gene sequence revealed, strain TW-1^T^ formed a lineage within the family *Oxalobacteraceae* and clustered as members of the genus *Massilia*. The closest members were *M. pinisoli* T33^T^ (98.8% sequence similarity), *M. putida* 6NM-7^T^ (98.6%), *M. arvi* THG-RS2O^T^ (98.5%), *M. phosphatilytica* 12-OD1^T^ (98.3%) and *M. niastensis* 5516S-1^T^ (98.2%). The sole respiratory quinone is ubiquinone-8. The major cellular fatty acids are hexadeconic acid, *cis*-9, methylenehexadeconic acid, summed feature 3 and summed feature 8. The major polar lipids are phosphatidylethanolamine, diphosphatidylglycerol and phosphatidylglycerol. The DNA G + C content of the type strain is 66.3%. The average nucleotide identity (ANI) and in silico DNA–DNA hybridization (dDDH) relatedness values between strain TW-1^T^ and closest members were below the threshold value for species demarcation. The genome size is 7,051,197 bp along with 46 contigs and 5,977 protein-coding genes. The genome showed 5 putative biosynthetic gene clusters (BGCs) that are responsible for different secondary metabolites. Cluster 2 showed thiopeptide BGC with no known cluster blast, indicating TW-1^T^ might produce novel antimicrobial agent. The antimicrobial assessment also showed that strain TW-1^T^ possessed inhibitory activity against Gram-negative pathogens (*Escherichia coli* and *Pseudomonas aeruginosa*). This is the first report of the species in the genus *Massilia* which produces antimicrobial compounds. Based on the polyphasic study, strain TW-1^T^ represents novel species in the genus *Massilia*, for which the name *Massilia antibiotica* sp. nov. is proposed. The type strain is TW-1^T^ (= KACC 21627^T^ = NBRC 114363^T^).

## Introduction

Antimicrobial-resistance (AMR) is the massive public health threat in the world^1^. Continuously elevated number of multidrug-resistant (MDR) strains harden the efficient treatment of infections caused by bacteria^2^. The infections caused by MDR strains are tremendously tough to treat and might need last resort of antibiotics^1, 3^. On the other hand, bacteria have been developed AMR to all antibiotics discovered to date^1^ and no novel antibiotics have been reported since long period. A review by Jim O’Nill estimated 700,000 deaths annually due to MDR infections caused by bacteria^4^. Furthermore, a study by Naylor et al*.* guesstimated healthcare system costs more than $90 million per year globally^5^. These consequences exhibited that AMR is not only accountable for public health hurdle but also pondered as economic burden. Hence, search for formidable and new antibiotics for the treatment of MDR infections caused by bacteria is extremely required.

We are continuously reconnoitring the previously uncultivated bacteria with the hope that they might produce a new bioactive molecule that may have pharmaceutical applications and might bioremediate the recalcitrant hydrocarbons. During the study of searching oil-degrading bacteria, we have surprisingly isolated a novel candidate of the genus *Massilia* producing antimicrobial agent that hinders the growth of *Pseudomonas aeruginosa* and *Escherichia coli*. The bioactive molecules (antimicrobial agent) from Gram-negative bacteria are scarcely reported. On the contrary, almost all the antibiotics have been reported from Gram-positive bacteria such as *Streptomyces*^6^. In this context, the report of this novel strain which possesses antimicrobial activity seems valuable.

The genus *Massilia* was first proposed by La Scola et al.^7^ with the description of *Massilia timonae*, which was isolated from blood of immunocompromised patient with cerebral lesion. Subsequently, the genus description has been emended by Kämpfer et al.^8^ and Singh et al*.*^9^. To date, 47 species of the genus *Massilia* with validly published names have been reported (https://lpsn.dsmz.de/genus/massilia). Members of the genus *Massilia* are characterized by Gram-stain-negative, rod-shaped and contain ubiquinone-8 (Q-8) as predominant isoprenoid quinone; C_16:0_, cyclo-C_17:0_, summed feature 3 (iso-C_15 :0_ 2-OH/C_16 :1_*ω*7*c*), summed feature 8 (C_18:1_*ω*6*c* and/or C_18 :1_*ω*7*c*) and C_14:0_ as major fatty acids; phosphatidylethanolamine (PE), diphosphatidylglycerol (DPG) and phosphatidylglycerol (PG) as a principal polar lipids^8–12^. Although most members of the genus *Massilia* have been isolated from soil, others have been isolated from air, drinking water, rock surface, ice-core, glacier permafrost and human clinical samples^7–21^. Some members have propensity to produce bioactive substance such as dimethyl disulfide (DMDS), which could have potential for controlling plant foliar diseases^10^. In addition, *M. chloroacetimidivorans* has been reported to degrade chloroacetamide herbicide^22^. On the other hand, some species such as *M. timonae*, *M. consociate*, *M. oculi* and *M. haematophila* are considered as pathogenic strains as they were isolated from human clinical samples^7, 8, 18^. However, to our best understanding, no member of the genus *Massilia* has been reported yet to possess antimicrobial activity.

In this study, a novel member of the genus *Massilia* isolated from oil-contaminated experimental soil having promising antimicrobial activity against *E. coli* and *P. aeruginosa* (Gram-negative pathogens) has been described with its phylogenetic and taxonomic position. In addition, whole-genome analysis of strain TW-1^T^ has been explored providing deeper insights into metabolic products.

## Materials and methods

### Isolation and preservation

Strain TW-1^T^ was isolated unexpectedly during the bioremediation experiment from oil-contaminated natural soil. The oil-contaminated soil was collected form industrial site located near Jeonju City, Republic of Korea. Isolation, maintenance and preservation of strain TW-1^T^ was carried as mentioned in previous study^23^.

### Phylogenetic analysis

Genomic DNA of strain TW-1^T^ was isolated by using InstaGene Matrix kit (Life Science Research; Bio-Rad) following manufacturer’s instruction. The 16S rRNA gene was amplified by using PCR (Bio-Rad) with forward and reverse primers 27F and 1492, respectively^24^. Applied Biosystems 3770XL DNA analyzer was used with a BigDye Terminator cycle sequencing Kit v.3.1 (Applied Biosystems, USA) for gene sequencing. After sequencing, nearly complete sequence of 16S rRNA genes was assembled using SeqMan software (DNASTAR Inc., USA). Phylogenetically closest neighbours were identified using the EzBioCloud server^25^ and ncbi megablast. All the 16S rRNA gene sequences of phylogenetically closest neighbours were retrieved from the ncbi GenBank database. All the retrieved sequences along with TW-1^T^ were aligned using in silico by silva alignment (https://www.arb-silva.de/aligner/). Neighbor-joining (NJ), maximum-likelihood (ML) and maximum-parsimony (MP) phylogenetic trees were reconstructed using mega (v7.0.26) software^26^.

### Genome analyses

For genome sequencing, extraction of genomic DNA was carried out by using DNeasy Blood and Tissue kits (Qiagen). Whole-genome shotgun sequencing of strain TW-1^T^ was accomplished at Macrogen (Republic of Korea) using the Illumina HiSeq 2500 platform using a 150-bp × 2 paired-end kit. The whole-genome sequences were assembled by SPAdes (v3.2)^27^. The authenticity and legitimacy of the assembled genome were checked by comparing 16S rRNA gene sequence of strain TW-1^T^ using ncbi Basic Local Alignment Search Tool (blastn)^28^. Potential contamination of genome assembly was examined in silico by ContEst16S algorithm using EzBioCloud server (https://www.ezbiocloud.net/tools/contest16s)^29^. Then, the whole-genome sequence of strain TW-1^T^ was annotated using the ncbi PGAP (Prokaryotic Genome Annotation Pipeline; https://www.ncbi.nlm.nih.gov/genome/annotation_prok)^30^ and RAST (Rapid Annotation using Subsystem Technology; https://rast.nmpdr.org) server^31^. All the genome sequences of reference strains were retrieved from ncbi database. The DNA G + C content of strain TW-1^T^ and other references used in this study were calculated based on respective whole-genome sequences. Genome-based relatedness between TW-1^T^ and phylogenetically closest neighbours were determined based on ANI (Average Nucleotide Identity) in silico by OrthoANIu (https://www.ezbiocloud.net/tools/ani) algorithm^32^. The phylogenomic tree was reconstructed in silico using concatenated alignment of 92 core genes with UBCGs software^33^. Digital DNA-DNA hybridization (dDDH) was calculated in silico by the Genome-to-Genome Distance Calculator (GGDC 2.1) using the blast method^34^. The conventional DNA-DNA hybridization (DDH) was measured fluorometrically using photobiotin-labelled DNA probes and microdilution plates as recommended by Ezaki et al*.*^35^. Graphical circular map was constructed by using CGView (http://cgview.ca) server^36^. Transfer RNAs (tRNAs) and ribosomal RNAs (rRNAs) were analysed using tRNAscan-SE (http://lowelab.ucsc.edu/tRNAscan-SE)^37^ and rnammer (http://www.cbs.dtu.dk/services/RNAmmer)^38^ servers. The CRISPR genes and Cas clusters were determined in silico using the CRISPRCasFinder (https://crisprcas.i2bc.paris-saclay.fr) server. The anti-SMASH server was used to identify the biosynthetic gene clusters (BGCs) for various secondary metabolites^39^. The Clusters of Orthologous Group (COG) functional categories were allocated by digging against the KEGG (Kyoto Encyclopedia of Genes and Genomes) database^40^.

### Physiological analyses

The cell morphology of strain TW-1^T^, grown on R2A agar plate for 4–5 days at 28 °C were observed by using TEM (transmission electron microscopy; Talos L120C; FEI). The colony morphology of strain of TW-1^T^ was seen using a Zoom Stereo Microscope (SZ61; Olympus, Japan). Gram staining was performed as described previously^41^. The cell motility was examined in R2A (Reasoner’s Agar No. 2; MB cell; KisanBio) medium consisting 0.4% agar. Oxidase and catalase activities of strain TW-1^T^ was examined using 1% tetra-methyl-*p*-phenylenediamine dihydrochloride and 3% (v/v) H_2_O_2_, respectively. Growth at various temperatures (0–45 °C) on R2A agar plates was monitored for 10 days. Growth was observed on various media including brain heart infusion agar (BHI; Oxoid), Luria–Bertani agar (LBA; Oxoid), marine agar 2216 (Becton), nutrient agar (NA; Oxoid), R2A agar, sorbitol MacConkey agar (MA; Oxoid), tryptone soya agar (TSA; Oxoid), and veal infusion agar (VIA; Becton). DNase activity of strain TW-1^T^ was examined by using DNase agar (Oxoid). Tolerance of salt was checked in R2A broth supplemented with NaCl [Duksan Chemicals, Republic of Korea; 0–5% (w/v) at 0.5% interval]. The pH range was observed at 28 °C in R2A broth (pH 4–12 in increments of 0.5 pH units). Testing of pH after sterilization showed only minor changes. To analyse the optimum temperature, pH and NaCl, the growth curve was determined by measuring growth absorbance at 600 nm using a spectrophotometer (Biochrome Libra S4). Hydrolysis of Tweens 80, 60 and 40 were analysed as described by Smibert & Krieg^42^. The anaerobic growth of strain TW-1^T^ was observed for 10 days on R2A agar at 28 °C with BD GasPak™ EZ Gas Generating Pouch System (BD). Hydrolysis of casein CM-cellulose, starch and tyrosine were assessed as mentioned in previous study^43^. Production of H_2_S and indole was checked in SIM (sulfide indole motility medium; Oxoid). Malachite green was used for spore staining. Other physiological tests were examined by using API 20NE and API ID 32GN kits (bioMérieux). The enzyme activities of strain TW-1^T^ and other references were examined by using an API ZYM kit (bioMérieux) following the manufacturer’s instructions. All the biochemical tests including API were performed in duplicate.

For the determination of fatty acids, cells of reference strains and TW-1^T^ were harvested from identical culture condition (at 28 °C for 4 days). Fatty acid methyl esters (FAME) of harvested cells were extracted using MIDI protocol technical note #101 (http://midi-inc.com/pdf/MIS_Technote_101.pdf). Extracted FAMEs were analysed using a HP 6890 Series GC System (Gas chromatograph; Hewlett Packard; Agilent Technologies) and the FAME compositions (percentage of totals) were identified with TSBA6 database of the Microbial Identification System^44^. The polar lipids and isoprenoid quinones were extracted from freeze-dried cells following the protocol of Minnikin et al.^45^. Isoprenoid quinone was analysed by using the HPLC (Agilent 1200 series) with following conditions. Solvent system, acetonitrile: iso-propanol (65: 35); flow rate, 1.2 mL/min; detection, 270 nm; run time, 20 min; and injection volume, 20 µL. Appropriate reagents for the spot detection were used as given by Komagata and Suzuki^46^.

### Antimicrobial activities of strain TW-1^T^

Antimicrobial activities of strain TW-1^T^ against *E. coli* KACC 10,185 and *P. aeruginosa* KEMB 121–234 were examined by disc-diffusion and spotting method, respectively. Screening were done against these Gram-negative pathogens by spotting the colonies of strain TW-1^T^ on R2A agar plates and incubated at 28 °C for 48 h. Crude product of culture extract was prepared by the culture supernatant of strain TW-1^T^ to evaluate disc-diffusion test. Strain TW-1^T^ was cultured in 300 mL of R2A broth at 28 °C (180 rpm for 5 days) into a 500 mL Erlenmeyer flask. Culture supernatant of strain TW-1^T^ was extracted by equal volume of ethyl acetate (2 ×) with pH 2.0 and 10, respectively^47^. Organic layer collected from extraction was completely evaporated by Rotary evaporator, (Eyela) and remained residue of crude product was dissolved in 500 µL of methanol. Then, 15 µL of crude product was diffused to a paper-disc (6 mm, Whatman) and antimicrobial activities against *P. aeruginosa* and *E. coli* KEMB were checked by measuring the inhibition zones. Trimethoprim/sulfamethaxazole (15 µg) and only methanol (15 µL) were used for positive and negative controls, respectively.

### Ethics approval

This study does not describe any experimental work related to human.

## Result and discussions

### Phylogenetic analysis

**Figure 1:**
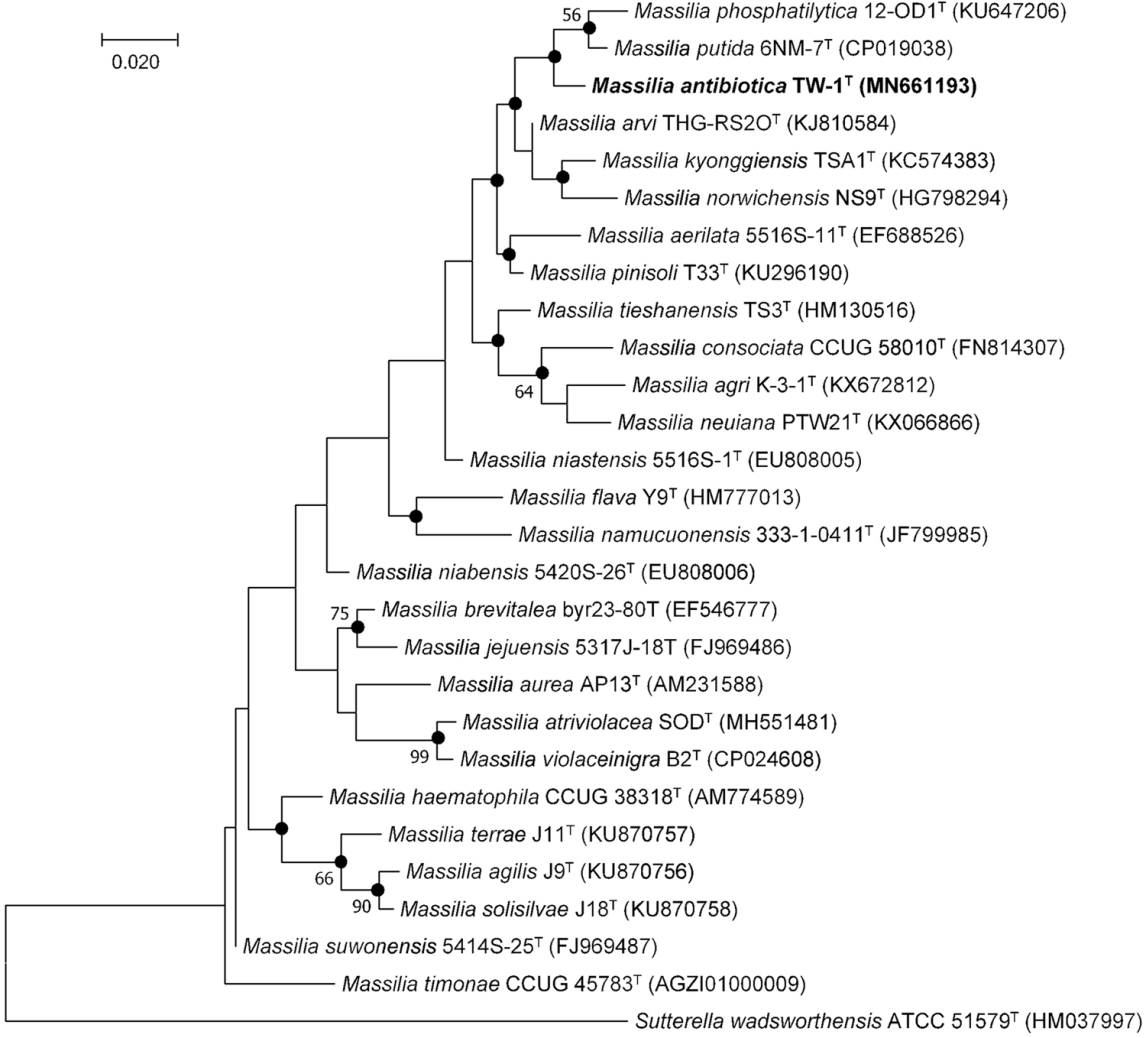
Maximum-likelihood phylogenetic tree based on 16S rRNA gene sequences showing the position of strain TW-1^T^ among the closest members of the genus *Massilia*. Filled circles are recovered by neighbour–joining and maximum–likelihood treeing methods. Bootstrap values (> 50%) based on 1000 bootstrap replicates are shown at branch nodes. *Sutterella wadsworthensis* ATCC 51579^T^ was used as an out-group. Numbers in parentheses indicate GenBank accessions. Bar, 0.020 substitutions per nucleotide position.

The 16S rRNA gene sequence of strain TW-1^T^ was 1,480 bp long and has been deposited at GenBank/EMBL/DDBJ database under the accession MN661193. Phylogenetic analysis based on 16S rRNA gene sequence showed that strain TW-1^T^ formed a lineage within the family *Oxalobacteraceae* and clustered as members of the genus *Massilia*. Strain TW-1^T^ was closest to *M. pinisoli* T33^T^ (98.8% sequence similarity) followed by *M. putida* 6NM-7^T^ (98.6%), *M. arvi* THG-RS2O^T^ (98.5%), *M. phosphatilytica* 12-OD1^T^ (98.3%) and *M. niastensis* 5516S-1^T^ (98.2%). The sequence similarities between strain TW-1^T^ and other members of the genus *Massilia* were < 98%. Strain TW-1^T^ was well clustered with *M. putida*, and *M. phosphatilytica* in both NJ and ML tress and formed a distinguish lineage in MP phylogenetic trees (Figs. 1, S1 and S2). The separate lineage formed in between *M. putida* and *M. phosphatilytica* in MP tree strongly supported the identification of strain TW-1^T^ as a novel member in the genus *Massilia* (S2).

### Genomic analysis

**Figure 2:**
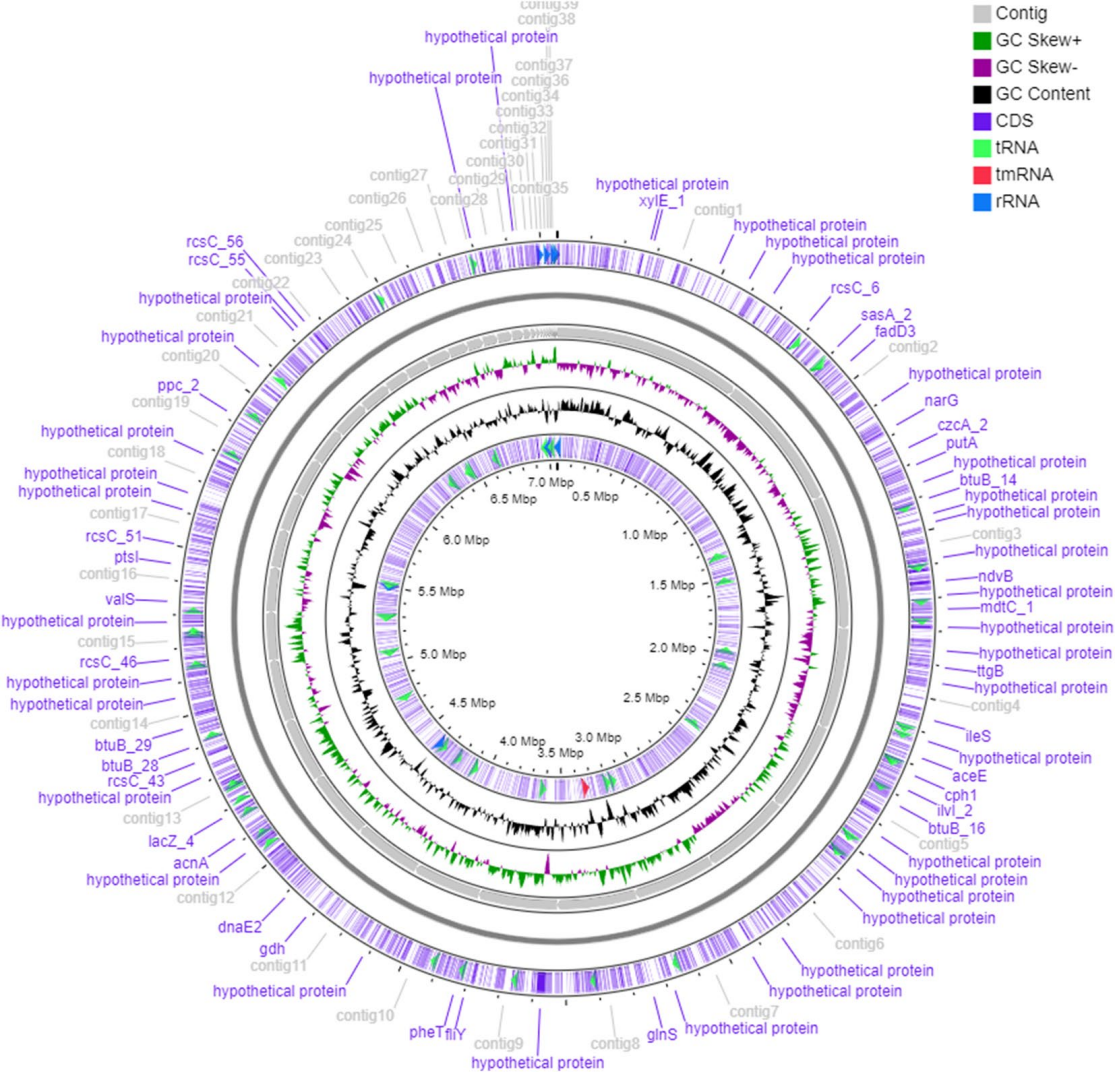
Graphical genomic map of strain TW-1^T^.

**Figure 3:**
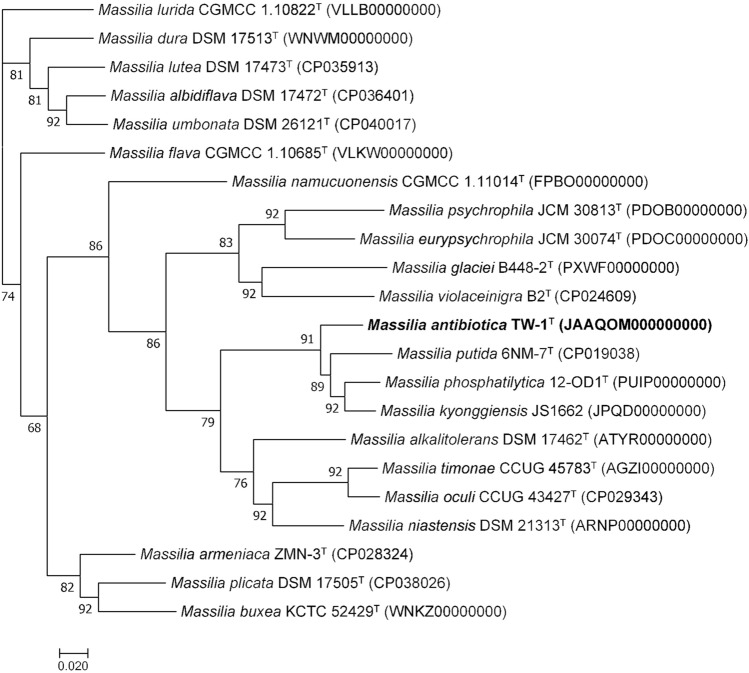
Phylogenomic tree constructed using UBCGs (concatenated alignment of 92 core genes). Bar, 0.020 substitution per nucleotide position.

The ContEst16S analysis showed that the genome belonged to strain TW-1^T^ and the genome has not been contaminated. Whole-genome shotgun sequence has been deposited at DDBJ/ENA/GenBank under the accession JAAQOM000000000. The genome size and N50 value of strain TW-1^T^ are 7,051,197 bp and 309,394 bp, respectively. The genome has 46 contigs and coverage of 89.0 × (Table S1). The graphical genomic map revealed the presence of 12 rRNAs (Fig. 2). The DNA G + C content of strain TW-1^T^ is 66.3% and within the range of *Massilia* species^8^. The ANI threshold for species delineation is recommended at 95–96%^48^ and ANIu between strain TW-1^T^ and phylogenetically closest neighbours are ≤ 87.8% (Table 1). The dDDH values of ≤ 34.4% is much lower than the species threshold of 70% recommended for species demarcation^34^ (Table 1). Moreover, DDH relatedness between strains TW-1^T^, *M. pinisoli* KACC 18748^T^ and *M. arvi* KACC 21416^T^ were 40.2 ± 2.6 and 24.1 ± 2.1%, respectively. These data clearly show that strain TW-1^T^ represents a novel member within the genus *Massilia*^34^. Furthermore, the phylogenomic tree constructed using UBCGs (concatenated alignment of 92 core genes) also proved that strain TW-1^T^ was a novel member of the genus *Massilia* (Fig. 3).

**Table 1:** Average nucleotide identity (ANIu) and digital DNA-DNA hybridization (dDDH) between strain TW-1^T^ and phylogenetically closest members of the genus *Massilia*.

Strains	Accessions	TW-1^T^	
		ANIu	dDDH
*Massilia albidiflava* DSM 17472^T^	CP036401	76.2	21.2
*Massilia alkalitolerans* DSM 17462^T^	ATYR00000000	78.0	21.5
*Massilia armeniaca* ZMN-3^T^	CP028324	76.5	21.2
*Massilia buxea* KCTC 52429^T^	WNKZ00000000	76.2	21.0
*Massilia dura* DSM 17513^T^	WNWM00000000	76.1	21.2
*Massilia eurypsychrophila* JCM 30074^T^	PDOC00000000	77.1	20.9
*Massilia flava* CGMCC 1.10685^T^	VLKW00000000	76.8	21.0
*Massilia glaciei* B448-2^T^	PXWF00000000	76.5	21.3
*Massilia kyonggiensis* JS1662	JPQD00000000	87.3	33.3
*Massilia lurida* CGMCC 1.10822^T^	VLLB00000000	76.5	20.9
*Massilia lutea* DSM 17473^T^	CP035913	76.1	20.9
*Massilia namucuonensis* CGMCC 1.11014^T^	FPBO00000000	76.3	20.7
*Massilia niastensis* DSM 21313^T^	ARNP00000000	78.4	22.0
*Massilia oculi* CCUG 43427^T^	CP029343	77.5	21.5
*Massilia phosphatilytica* 12-OD1^T^	PUIP00000000	87.5	33.2
*Massilia plicata* DSM 17505^T^	CP038026	76.2	20.9
*Massilia psychrophila* JCM 30813^T^	PDOB00000000	76.2	20.4
*Massilia putida* 6NM-7^T^	CP019038	87.8	34.4
*Massilia timonae* CCUG 45783^T^	AGZI00000000	77.9	21.5
*Massilia umbonata* DSM 26121^T^	CP040017	76.5	21.3
*Massilia violaceinigra* B2^T^	CP024609	76.1	20.7

**Figure 4:**
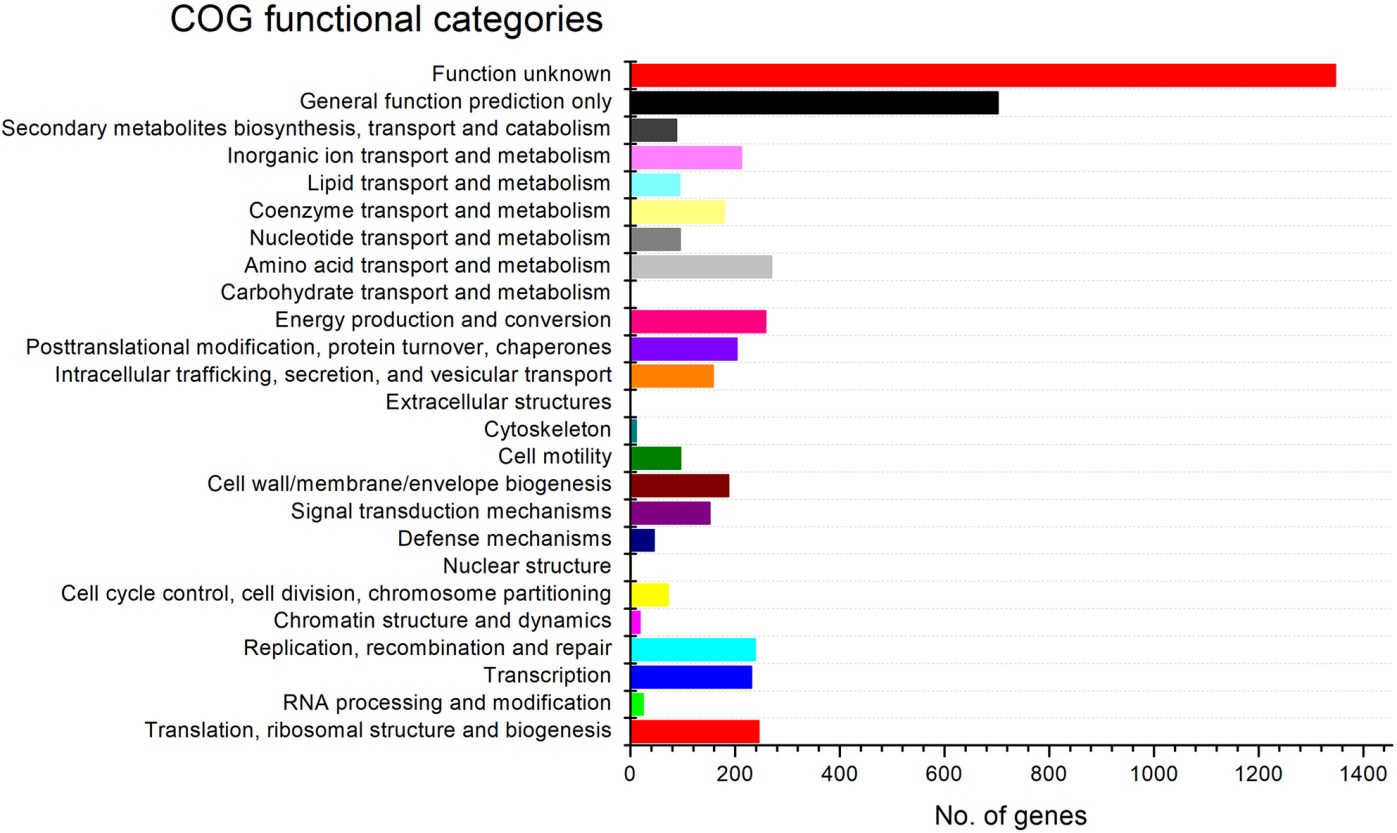
COG functional classification of proteins in the genome of strain TW-1^T^.

The RAST analysis revealed the presence of 339 subsystems and 4 secondary metabolisms consisting auxin biosynthesis (four, Fig. S3). The COG functional classification of proteins showed the highest and lowest number of genes were of unknown functions (1347) and extracellular structures (1) (Fig. 4). The genome of strain TW-1^T^ consists 5 putative BGCs (terpene, siderophore, bacteriocin, acyl_amino_acid and thiopeptide) were revealed by anti-SMASH analysis (Table 2). The core and additional biosynthetic genes were predicted along with gene flaking similarities for secondary metabolite biosynthetic gene clusters (smBGCs) (Table 2). Thiopeptide BGC showed only 33% of gene similarity with *Massilia putida* (NZ_CP019038; 4,695,767–4,724,913) with no known clusters. Additionally, each identified gene cluster from anti-SMASH analysis were compared against the ncbi database using protein–protein blast (BlastP) (Fig. S4). The core biosynthetic genes in the thiopeptide BGC showed identified nuclear transport factor 2 family protein and OsmC domain/YcaO domain-containing proteins (Fig. S4). As no known cluster has been predicted from the thiopeptide cluster, strain TW-1^T^ might produce unique natural products (Fig. S4). In addition, the genome contained three antibiotic biosynthesis monooxygenase (ABM; WP_166855736, WP_166862851 and WP_166864744) that are possibly responsible for biosynthesis for antibiotics.

**Table 2:** Numbers of predicted secondary metabolite biosynthetic gene clusters (smBGC) of TW-1^T^ genome. The BGCs were determined using anti-SMASH (v5.1.2).

Cluster	Gene flanking similarity (%)	smBGC type	From	To	Most similar known cluster	Core biosynthetic gene	Additional biosynthetic gene
1	94	Acyl_amino_acids	162,489	223,265	N-tetradecanoyl tyrosine (Other) (6%)	1	12
2	13	Thiopeptide	272,320	309,699	–	2	8
3	100	Bacteriocin	82,478	94,127	–	1	0
4	100	Terpene	106,679	128,373	–	2	1
5	75	Siderophore	32,740	44,710	–	1	1

Although strain TW-1^T^ was isolated at 28 °C, it could grow well at 4 °C. When we performed the genome mining of strain TW-1^T^ we found the genes (CspA, CspC) related to cold shock proteins and cold-shock domain containing proteins (WP_036166698, WP_166858078, WP_166858204, WP_056448893 and WP_03616538). These proteins help the organism to adapt in cold temperatures. The genome contained arsenic resistance gene arsH (WP_166857685) and chromate resistance gene (WP_166864147) showing the strain could tolerate arsenic and chromate. The genome contained various protease genes such as rhomboid family intramembrane serine protease (WP_166860501), site-2 protease (S2P) family protein (WP_166861383), ATP-dependent Clp protease ATP-binding subunit ClpX (WP_166861695), ATP-dependent protease ATPase subunit HslU (WP_166858142), ATP-dependent protease subunit HslV (WP_166857747), trypsin-like serine protease (WP_166857912, WP_166858974), protease HtpX (WP_166861379, WP_166859769), DJ-1/PfpI/YhbO family deglycase/protease (WP_166859796, WP_166864477, WP_166859797), ATP-dependent Clp protease adapter ClpS (WP_027864722), FtsH protease activity modulator HflK (WP_166865455), and protease modulator HflC (WP_166865458). Presence of these various proteases encoding genes indicate the industrial and medical significance of strain TW-1^T^. Bacterial proteases are widely used in the industrial sectors for various enzymatic activities and currently these enzymes are also regarded as valuable resources for antimicrobial drug targets^49, 50^.

### Physiological analysis

The cells of strain TW-1^T^ are rod-shaped (Fig. S5), Gram-stain-negative, aerobic and motile with polar flagellum. The indole test is negative and strain TW-1^T^ is non-spore-forming. Strain TW-1^T^ hydrolysed DNA, CM-cellulose, casein, aesculin, starch, Tweens 40 and 60. Hydrolysis of chitin is negative but weakly hydrolyse Tween 80, gelatin and tyrosine. Red diffusible pigmentation was also observed while hydrolysing tyrosine. Strain TW-1^T^ grew well but the references were unable to grow at 4 °C. Other differential physiological characteristics are given on Table 3 with phylogenetically closest species of the genus *Massilia*.

**Table 3:** Phenotypic characteristics of strain TW-1^T^ that differentiates with phylogenetically related species of the genus *Massilia*.

Characteristic	1	2	3	4	5	6	7
Highest growth temperature (°C)	42	37	37	37	42	40	42
pH range	4.5–10.5	5.5–9.0	5.5–8.5	5.0–9.0	6.0–8.5	5.5–9.0	5.0–9.0
Highest NaCl tolerance (%, w/v)	1	1	0.5	0.5	0.5	1	2
Nitrate reduction	+	+	–	+	+	–	+
Urease activity	–	–	–	–	+	–	–
**Hydrolysis of**							
Aesculin	+	+	–	–	+	+	+
Casein	+	–	–	–	–	–	+
Gelatin	w	–	+	–	–	+	–
Starch	+	–	+	–	+	+	+
**Enzyme activity**							
Acid phosphatase	+	–	–	–	+	+	+
Alkaline phosphatase	+	+	+	+	+	+	w
Cystine arylamidase	+	+	+	–	+	+	–
Esterase (C4)	–	+	+	+	+	+	w
Esterase lipase (C8)	w	+	+	+	+	–	–
Leucine arylamidase	+	–	–	+	+	+	+
Lipase (C14)	w	–	–	–	–	–	–
*N*-acetyl-*β*-glucosaminidase	+	–	–	–	+	–	+
Valine arylamidase	+	–	–	+	+	+	+
*α*-galactosidase	+	+	+	+	+	–	+
*α*-glucosidase	+	+	+	–	+	–	+
*β*-galactosidase	+	+	–	–	+	+	w
*β*-glucuronidase	w	–	–	–	–	–	–
**Assimilation from**							
2-Ketogluconate	–	–	–	+	–	–	–
3-Hydroxybenzoic acid	–	–	–	–	–	+	–
3-Hydroxybutyric acid	+	–	–	–	–	+	w
4-Hydroxybenzoic acid	+	–	–	–	–	+	–
Adipic acid	–	–	–	+	–	–	w
Capric acid	–	–	–	–	w	–	–
d-maltose	+	–	+	+	+	+	+
d-mannose	+	–	+	+	+	+	+
d-melibiose	+	–	–	–	–	–	–
d-saccharose (sucrose)	+	+	+	+	–	–	+
Glycogen	+	+	–	–	–	–	+
Lactic acid	+	–	–	–	–	–	w
l-alanine	+	–	–	–	–	+	–
l-arabinose	+	–	+	+	+	+	+
l-fucose	+	–	–	+	–	–	+
l-histidine	+	–	–	–	–	–	–
l-proline	w	–	+	–	–	+	–
l-rhamnose	+	–	–	–	+	+	+
Malic acid	–	–	+	+	–	+	+
*N*-acetyl-glucosamine	–	+	–	–	+	+	–
Phenylacetic acid	–	–	–	+	–	–	+
Potassium gluconate	–	–	+	+	+	+	w
Salicin	–	–	–	–	–	+	–
Sodium acetate	–	–	–	–	–	+	–
Trisodium citrate	–	–	–	+	w	–	w
DNA G + C content (mol%)	66.3	69.4*	64.7	66.2	63.2†	66.3	66.7‡

Strains: 1, TW-1^T^; 2, *M. pinisoli* KACC 18748^T^; 3, *M. putida* KACC 21418^T^; 4, *M. phosphatilytica* KACC 21417^T^; 5, *M. arvi* KACC 21416^T^; 6, *M. niastensis* KACC 12599^T^; 7, *M. kyonggiensis* KACC 17471^T^. All data were obtained from this study except otherwise indicated. + , positive; w, weak; –, negative.

Data from *Altankhuu and Kim^12^; †Singh et al*.*^9^; and ‡Kim^21^.

The major fatty acids of strain TW-1^T^ were C_16:0_, cyclo-C_17:0_, summed feature 3 (iso-C_15 :0_ 2-OH/C_16 :1_*ω*7*c*), summed feature 8 (C_18:1_*ω*6*c* and/or C_18 :1_*ω*7*c*) and C_14:0_, similar with the genus *Massilia*. The differences in major and minor fatty acids in addition to the presence of minor fatty acid, cyclo-C_19:0_ *ω*8*c* differentiate strain TW-1^T^ from other phylogenetically related species of the genus *Massilia* (Table 4). The sole respiratory quinone was ubiquinone-8 (Q-8) and the major polar lipids were phosphatidylethanolamine, diphosphatidylglycerol and phosphatidylglycerol. In addition, two unidentified phospholipids (PL1–PL2), three unidentified aminolipids (AL1-AL3) and three unidentified polar lipids (L1-L3) are also seen in TLC chromatogram (Fig. S6).

**Table 4:** Cellular fatty acid profiles (percentage of totals) of TW-1^T^ and phylogenetically related reference strains of the genus *Massilia*.

Fatty acid	1	2	3	4	5	6	7
**Saturated**							
C_10:0_	0.6	–	–	0.9	1.5	–	–
C_12:0_	2.4	2.8	2.0	4.5	–	4.7	4.8
C_14:0_	6.4	5.1	4.3	4.7	4.4	1.0	3.9
C_16:0_	31.3	24.7	33.3	24.1	28.9	26.1	27.6
C_18:0_	0.2	–	–	–	1.5	–	–
**Hydroxy**							
C_12:0_ 2-OH	–	–	–	–	–	1.9	–
C_14:0_ 2-OH	1.2	–	2.7	6.2	2.9	0.6	2.9
C_10:0_ 3-OH	3.5	3.4	5.9	9.8	6.1	5.3	–
**Branched saturated**							
Cyclo-C_17:0_	22.7	3.9	20.9	25.0	10.7	2.9	7.1
Cyclo-C_19:0_ *ω*8*c*	0.6	–	–	–	–	–	–
Summed features*							
3	20.0	48.7	21.9	22.5	33.3	44.4	43.1
8	9.6	11.4	7.4	1.7	8.9	11.1	10.0

Strains: 1, TW-1^T^; 2, *M. pinisoli* KACC 18748^T^; 3, *M. putida* KACC 21418^T^; 4, *M. phosphatilytica* KACC 21417^T^; 5, *M. arvi* KACC 21416^T^; 6, *M. niastensis* KACC 12599^T^; 7, *M. kyonggiensis* KACC 17471^T^. All data were obtained from this study. Fatty acid amounting < 0.5% in all strains are not listed. –, not detected.

*Summed features represent groups of two or three fatty acids that could not be separated using the MIDI system. Summed feature 3 comprised iso-C_15 :0_ 2-OH/C_16 :1_*ω*7*c* and summed feature 8 comprised C_18:1_*ω*6*c* and/or C_18 :1_*ω*7*c*.

### Antimicrobial activities

Strain TW-1^T^ showed antimicrobial activities against Gram-negative pathogens. The zone of inhibitions for *P. aeruginosa* KACC 10,185 and *E. coli* KEMB 121–234 were 17 and 18 mm, respectively (Fig. S7). This is unique result that we have isolated the bacterial strain having potent antimicrobial effects against Gram-negative pathogens from oil-contaminated soil and we report this is the first study of *Massilia* species producing antimicrobial compound. The determination of MIC value in addition to identification and characterization of bioactive compound are under investigation. However, based on anti-SMASH analysis, there is a high chance to get a novel antimicrobial compound from strain TW-1^T^ as it showed thiopeptide smBGC with no known cluster blast (Fig. S4).

Based on above discussed data, strain TW-1^T^ represents a novel species within the genus *Massilia* for which the name *Massilia antibiotica* sp. nov. is proposed.

### Description of *Massilia antibiotica* sp. nov.

*Massilia antibiotica* (an.ti.bi.o’ti.ca. Gr. pref. *anti* against; Gr. masc. n. *bios* -life; N.L. fem. adj. *antibiotica* against life, antibiotic).

Cells (2.9–3.4 µm long and 0.9–1.0 µm wide) are rod-shaped, strictly aerobic, Gram-stain-negative and motile with polar flagellum. Colonies grow well on R2A agar plate and weakly on NA, while no growth is observed on BHI, LBA, MA, marine agar 2216, TSA and VIA. Colonies on R2A are ivory coloured, circular and convex. The colony size is 2–3 mm on R2A agar plate for 5 days at 28 °C. Colonies grow at 4–42 °C (optimum, 25–35 °C) and pH 4.5–10.5 (optimum pH, 6.5–9.0). The strain grows optimally in the absence of NaCl but tolerate 1% (w/v) of NaCl. Catalase and oxidase are positive. Hydrogen sulfide is not produced. Nitrate is reduced to nitrite. Glucose is not fermented. PNPG (4-nitrophenyl -*βd*-galactopyranoside) is positive. The type strain shows the following enzyme activities: positive for alkaline phosphatase, leucine arylamidase, valine arylamidase, cystine arylamidase, acid phosphatase, *α*-galactosidase, *β*-galactosidase, *α*-glucosidase, *β*-glucosidase and *N*-acetyl-*β*-glucosaminidase; weakly positive for esterase lipase (C8), lipase (C14), trypsin, *α*-chymotrypsin, napthol-AS-BI-phosphohydrolase and *β*-glucuronidase; and negative for esterase (C4), *α*-mannosidase and *α*-fucosidase. The following substrates are assimilated: 3-hydroxybutyric acid, 4-hydroxybenzoic acid, d-glucose, d-maltose, d-mannose, d-melibiose, glycogen, lactic acid, l-alanine, l-arabinose, l-fucose, l-histidine, l-proline and sucrose. The sole respiratory quinone is Q-8. The major cellular fatty acids are C_16:0_, cyclo-C_17:0_, summed feature 3 (iso-C_15 :0_ 2-OH/C_16 :1_*ω*7*c*), summed feature 8 (C_18:1_*ω*6*c* and/or C_18 :1_*ω*7*c*) and C_14:0_. The major polar lipid are phosphatidylethanolamine, diphosphatidylglycerol and phosphatidylglycerol. The DNA G + C content of the type strain is 66.3%.

The type strain, TW-1^T^ (= KACC 21627^T^ = NBRC 114363^T^), was isolated from oil-contaminated experimental soil in Kyonggi University, Republic of Korea. The GenBank/EMBL/DDBJ accession numbers for the 16S rRNA gene sequence and the whole genome sequence of strain TW-1^T^ are MN661193 and JAAQOM000000000, respectively.

## Supplementary Information

Supplementary Information
