# Author Correction: Genome insight and description of antibiotic producing *Massilia antibiotica* sp. nov., isolated from oil-contaminated soil

**DOI:** 10.1038/s41598-021-92164-5

**Published:** 2021-06-14

**Authors:** Ram Hari Dahal, Dhiraj Kumar Chaudhary, Jaisoo Kim

**Affiliations:** 1 Department of Life Science, College of Natural Sciences, Kyonggi University, Suwon, Kyonggi‑Do 16227 Republic of Korea; 2 Department of Microbiology, School of Medicine, Kyungpook National University, Daegu, 41944 Republic of Korea; 3 Department of Environmental Engineering, Korea University Sejong Campus, Sejong City, 30019 Republic of Korea

Correction to: *Scientific Reports* https://doi.org/10.1038/s41598-021-86232-z, published online 23 March 2021

The original version of this Article contained an error in the Result and discussions section, under the subheading ‘Description of *Massilia antibiotica* sp. nov.’, where,

“The type strain, TW-1^T^ 1^T^ (= KACC 21627^T^ = NBRC 114363^T^), was isolated from oil-contaminated experimental soil in Kyonggi University, Republic of Korea.”

now reads:

“The type strain, TW-1^T^ (= KACC 21627^T^ = NBRC 114363^T^), was isolated from oil-contaminated experimental soil in Kyonggi University, Republic of Korea.”

The original Article has been corrected.

