# Supplementary material for: Genome insight and description of antibiotic producing Massilia antibiotica sp. nov., isolated from oil-contaminated soil
Source: Sci Rep. 2021 Mar 23;11:6695. doi: 10.1038/s41598-021-86232-z (PMC7988111; doi:10.1038/s41598-021-86232-z)
Supplement: Supplementary file 1 — Supplementary Information [file 41598_2021_86232_MOESM1_ESM.pdf]

Supplementary Materials

Scientific Reports

Genome insight and description of antibiotic producing *Massilia antibiotica* sp. nov., isolated from oil-contaminated soil

Ram Hari Dahal[1,2], Dhiraj Kumar Chaudhary[3] and Jaisoo Kim[2]*

[1]Department of Microbiology, School of Medicine, Kyungpook National University, Daegu 41944, Republic of Korea

[2]Department of Life Science, College of Natural Sciences, Kyonggi University, Suwon, Kyonggi-Do 16227, Republic of Korea

[3]Department of Environmental Engineering, Korea University Sejong Campus, Sejong City 30019, Republic of Korea

Running title: Genome insight and description of *Massilia antibiotica*

Key words: *Massilia antibiotica*, Oil-contaminated soil, Multidrug-resistant, Antibiotics, Antimicrobial-resistance

Contents category: New Taxa; Subsection: *Proteobacteria*

*Corresponding author: Jaisoo Kim.

Tel: +82-31-249-9648; Fax: +82-31-253-1165; E-mail: jkimtamu@kgu.ac.kr

Supplementary Table S1. The genome features of strain TW-1^T.

Genome features	Value
Genome size (bp)	7,051,197
G+C content (mol%)	63.3
No. of contigs	46
N50	309,394
No. of subsystem	339
No. of proteins	5,977
Total genes	6,158
CDSs (total)	6,066
Protein-coding genes	5,977
Genes (RNA)	92
rRNAs (5S, 16S, 23S)	12 (6, 3, 3)
tRNAs	76
ncRNAs	4
Pseudo Genes (total)	89
CRISPR repeats	6
Cas cluster	6
Genome coverage	89.0×

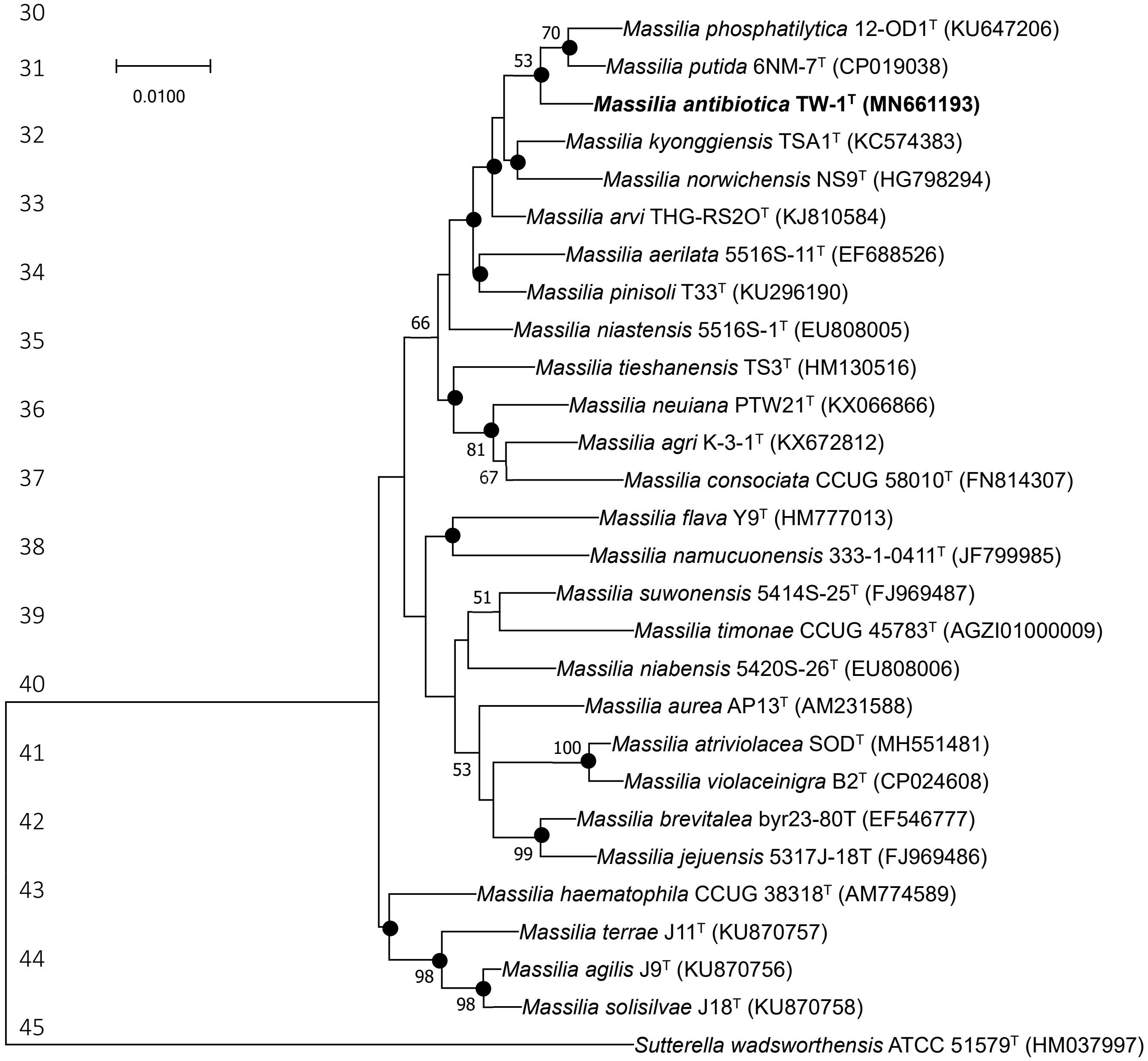

Supplementary Figure S1 Neighbour-joining tree based on 16S rRNA gene sequences showing the phylogenetic position of strain TW-1^{T} among the closely related members of the genus *Massilia*. Filled circles indicate nodes recovered by treeing methods (neighbour–joining and maximum–likelihood). The numbers at the nodes indicate the percentage of 1000 bootstrap replicates yielding this topology; only values >50% are shown. *Sutterella wadsworthensis* ATCC 51579^{T} was used as an out-group. GenBank accession numbers are given in parentheses. Bar, 0.010 substitutions per nucleotide position.

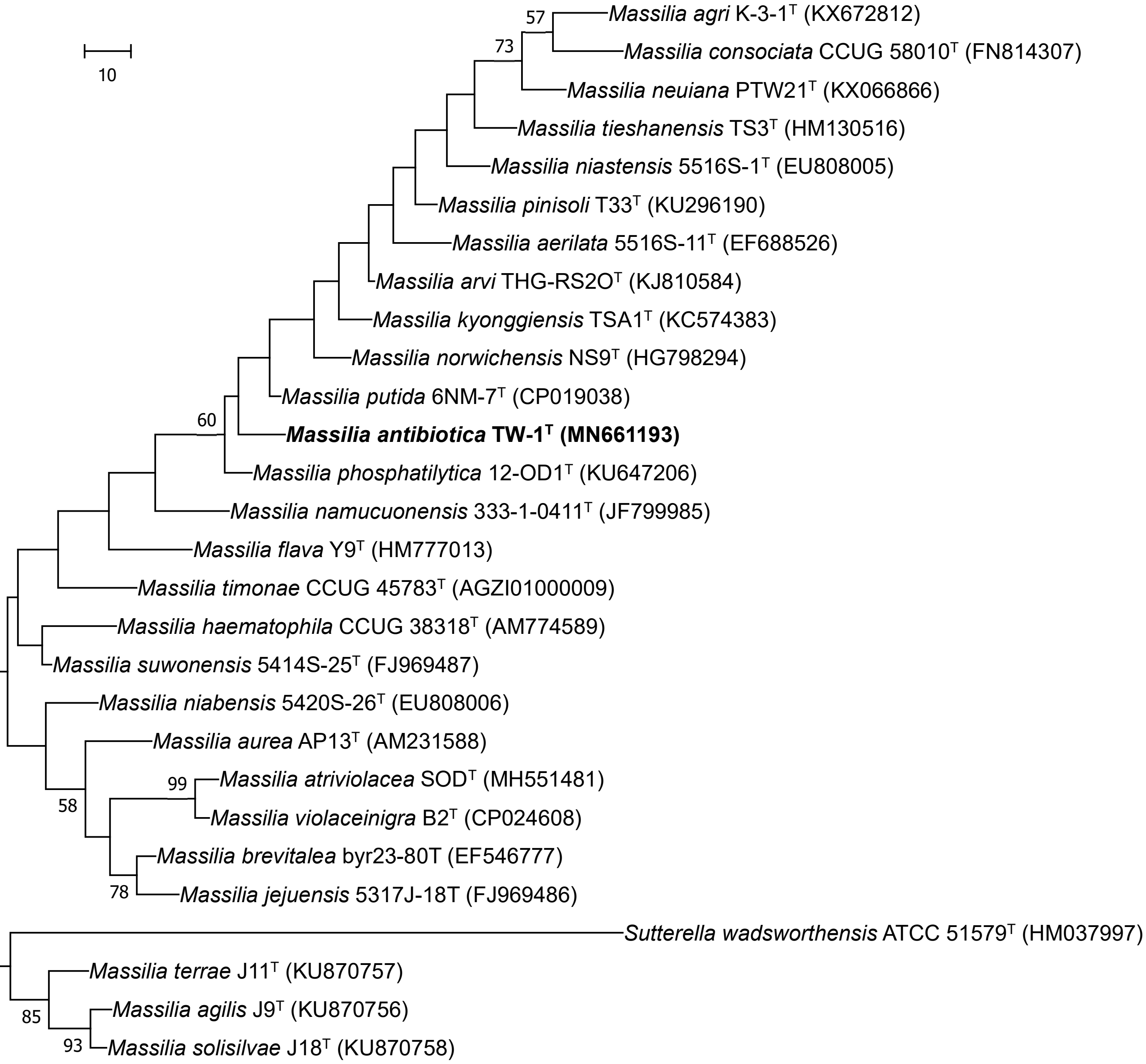

Supplementary Figure S2. Maximum-parsimony tree based on 16S rRNA gene sequences showing the phylogenetic position of strain TW-1^T among the closely related members of the genus *Massilia*. The numbers at the nodes indicate the percentage of 1000 bootstrap replicates yielding this topology; only values >50% are shown. *Sutterella wadsworthensis* ATCC 51579^T was used as an out-group. GenBank accession numbers are given in parentheses. Bar, 10 substitutions per nucleotide position.

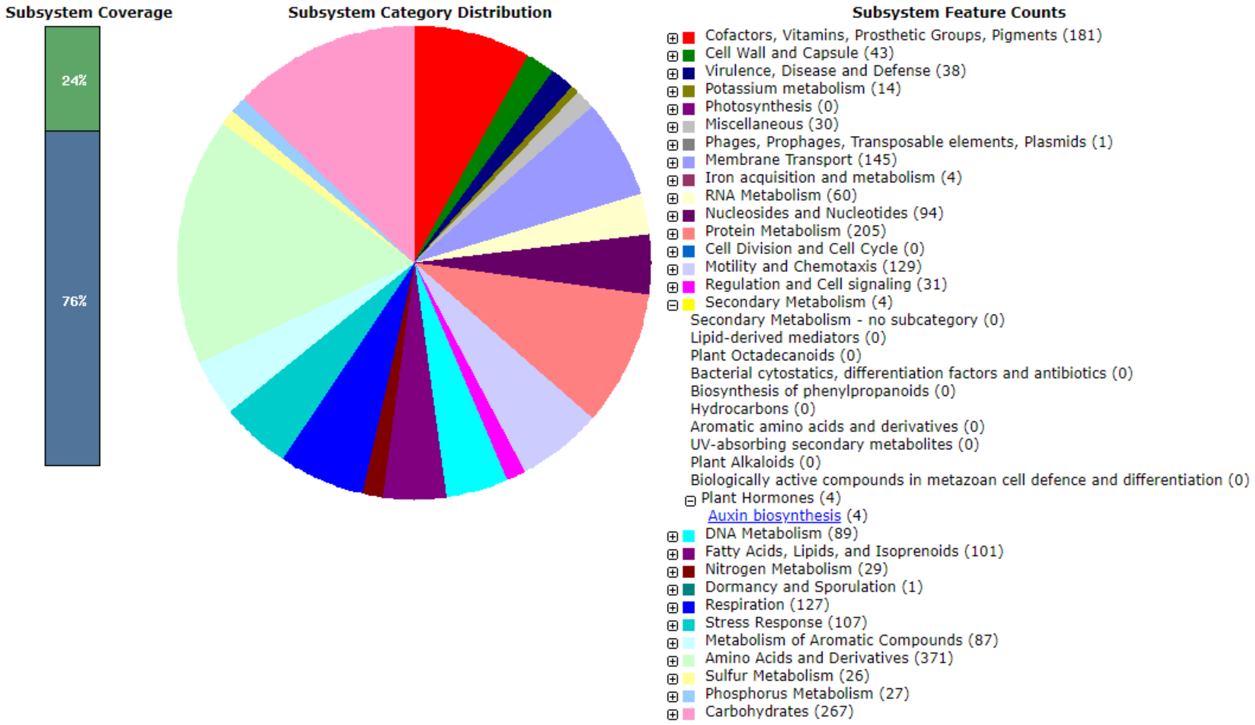

Supplementary Figure S3. Subsystem feature of strain TW-1^T revealed by RAST (Rapid Annotation using Subsystem Technology) server.

Supplementary Figure S4. Graphs of identified gene clusters obtained from anti-SMASH. Each gene was compared with the NCBI database employing BlastP (protein-protein BLAST). The colour code and the order correspond to the anti-SMASH prediction. Query sequence: strain TW-1^{T}. Maroon heighted rows showed core biosynthetic genes and pink highlighted gens showed additional biosynthetic genes.

Cluster 1: Acyl_amino_acids

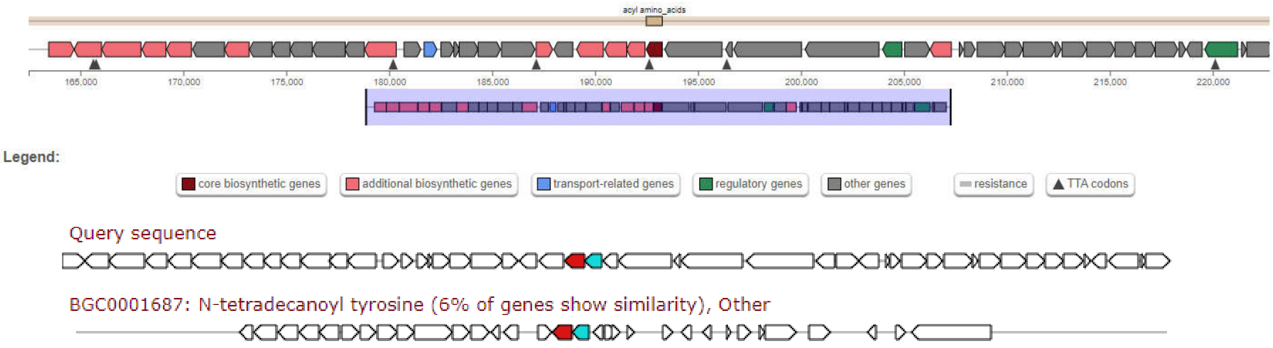

Contig No./locus tag	Identified protein name
Ctg3_145	Pyridoxal-dependent decarboxylase, exosortase A system-associated
Ctg3_146	Putative O-glycosylation ligase, exosortase A system-associated
Ctg3_147	Amidotransferase 1, exosortase A system-associated
Ctg3_148	TIGR03088 family PEP-CTERM/XrtA system glycosyltransferase
Ctg3_149	Glycosyltransferase, exosortase A system-associated
Ctg3_150	Exosortase A
Ctg3_151	TIGR03087 family PEP-CTERM/XrtA system glycosyltransferase
Ctg3_152	FemAB family PEP-CTERM system-associated protein
Ctg3_153	DUF3473 domain-containing protein
Ctg3_154	AAA family ATPase
Ctg3_155	TIGR03016 family PEP-CTERM system-associated outer membrane protein
Ctg3_156	AAA family ATPase
Ctg3_157	Chain length-determining protein
Ctg3_158	Trypsin-like peptidase domain-containing protein
Ctg3_159	Polysaccharide biosynthesis/export family protein
Ctg3_160	Hypothetical protein
Ctg3_161	HPr-rel-A system PqqD family peptide chaperone
Ctg3_162	HprK-related kinase A
Ctg3_163	Nucleotidyltransferase family protein
Ctg3_164	GMC family oxidoreductase N-terminal domain-containing protein
Ctg3_165	SDR family NAD(P)-dependent oxidoreductase
Ctg3_166	PEP-CTERM sorting domain-containing protein
Ctg3_167	Glycosyltransferase
Ctg3_168	Molybdopterin biosynthesis protein MoeY
Ctg3_169	ThiF family adenylyltransferase
**Ctg3_170**	**N-acetyltransferase**

Contig No./locus tag	Identified protein name
Ctg3_171	PEP-CTERM system TPR-repeat protein PrsT
Ctg3_172	Hypothetical protein
Ctg3_173	Cobalamin-dependent protein
Ctg3_174	Indolepyruvate ferredoxin oxidoreductase family protein
Ctg3_175	LysR family transcriptional regulator
Ctg3_176	DUF3391 domain-containing protein
Ctg3_177	Diguanylate cyclase
Ctg3_178	Flp family type IVb pilin
Ctg3_179	Prepilin peptidase
Ctg3_180	ATP-binding protein
Ctg3_181	Flp pilus assembly protein CpaB
Ctg3_182	Pilus assembly protein N-terminal domain-containing protein
Ctg3_183	Hypothetical protein
Ctg3_184	AAA family ATPase
Ctg3_185	Flp pilus assembly complex ATPase component TadA
Ctg3_186	Type II secretion system F family protein
Ctg3_187	Type II secretion system F family protein
Ctg3_188	LytR C-terminal domain-containing protein
Ctg3_189	BON domain-containing protein
Ctg3_190	DUF2807 domain-containing protein
Ctg3_191	Sigma 54-interacting transcriptional regulator
Ctg3_192	Hypothetical protein
Ctg3_193	RtcB family protein

Cluster 2: Thiopeptide

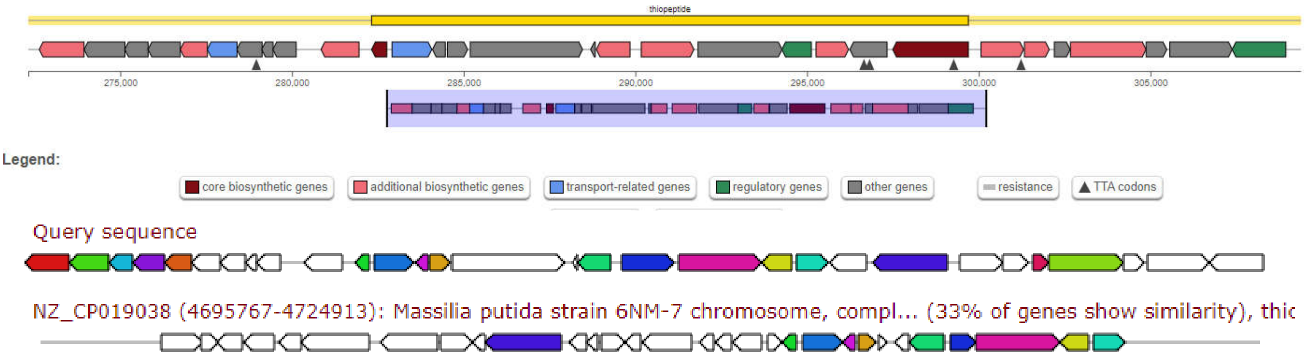

Contig No./locus tag	Identified protein name
Ctg7_237	Precorrin-6y C5,15-methyltransferase (decarboxylating) subunit CbiE
Ctg7_238	Cobalt-precorrin-5B (C(1))-methyltransferase
Ctg7_239	Precorrin-8X methylmutase
Ctg7_240	Sirohydrochlorin chelatase
Ctg7_241	Precorrin-4 C(11)-methyltransferase
Ctg7_242	ATP-binding cassette domain-containing protein
Ctg7_243	Cobalt ECF transporter T component CbiQ
Ctg7_244	Energy-coupling factor ABC transporter substrate-binding protein
Ctg7_245	Energy-coupling factor ABC transporter permease
Ctg7_246	N-ethylmaleimide reductase
**Ctg7_247**	**Nuclear transport factor 2 family protein**
Ctg7_248	ABC transporter substrate-binding protein
Ctg7_249	Hypothetical protein
Ctg7_250	YgjV family protein
Ctg7_251	DEAD/DEAH box helicase
Ctg7_252	Organic hydroperoxide resistance protein
Ctg7_253	Alpha/beta fold hydrolase
Ctg7_254	Carboxylesterase family protein
Ctg7_255	DUF4968 domain-containing protein
Ctg7_256	Helix-turn-helix domain-containing protein
Ctg7_257	Alpha/beta fold hydrolase
Ctg7_258	Hypothetical protein
**Ctg7_259**	**OsmC domain/YcaO domain-containing protein**
Ctg7_260	FAD-dependent monooxygenase
Ctg7_261	Thioredoxin fold domain-containing protein
Ctg7_262	2Fe-2S iron-sulfur cluster binding domain-containing protein
Ctg7_263	Molybdopterin-dependent oxidoreductase
Ctg7_264	NTP transferase domain-containing protein
Ctg7_265	PDZ domain-containing protein
Ctg7_266	Response regulator

Cluster 3: Bacteriocin

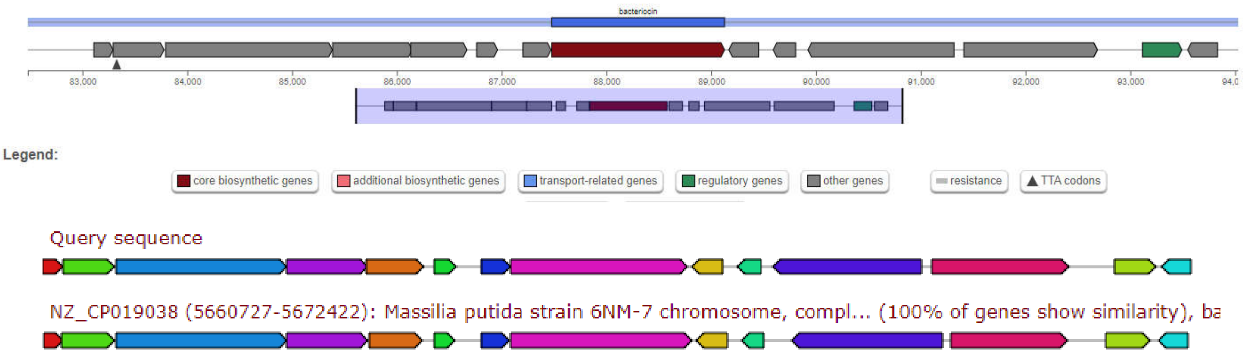

Contig No./locus tag	Identified protein name
Ctg8_74	Hypothetical protein
Ctg8_75	Hypothetical protein
Ctg8_76	AAA family ATPase
Ctg8_77	General secretion pathway protein GspB
Ctg8_78	Molybdopterin-guanine dinucleotide biosynthesis protein B
Ctg8_79	Putative motility protein
Ctg8_80	DUF2282 domain-containing protein
**Ctg8_81**	**DUF692 family protein**
Ctg8_82	Hypothetical protein
Ctg8_83	RNA-binding S4 domain-containing protein
Ctg8_84	Class II fumarate hydratase
Ctg8_85	Hypothetical protein
Ctg8_86	Winged helix-turn-helix transcriptional regulator
Ctg8_87	Flagellar protein FliT

Cluster 4: Terpene

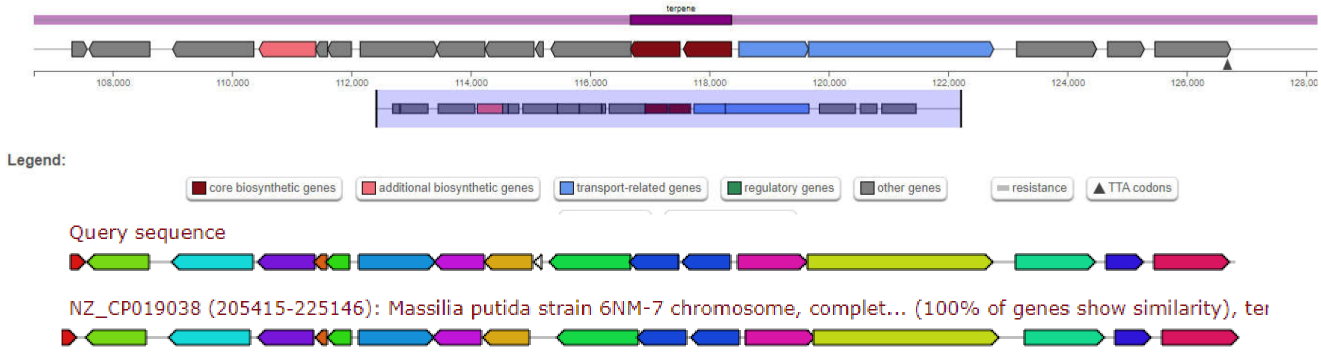

Contig No./locus tag	Identified protein name
Ctg13_89	Molybdopterin converting factor subunit 1
Ctg13_90	TRAP transporter substrate-binding protein DctP
Ctg13_91	M28 family peptidase
Ctg13_92	Alpha/beta fold hydrolase
Ctg13_93	Helix-turn-helix domain-containing protein
Ctg13_94	Hypothetical protein
Ctg13_95	TIGR03862 family flavoprotein
Ctg13_96	DUF2807 domain-containing protein
Ctg13_97	DUF1700 domain-containing protein
Ctg13_98	Hypothetical protein
Ctg13_99	FAD-dependent oxidoreductase
**Ctg13_100**	**Presqualene diphosphate synthase HpnD**
**Ctg13_101**	**Squalene synthase HpnC**
Ctg13_102	Efflux RND transporter periplasmic adaptor subunit
Ctg13_103	Efflux RND transporter permease subunit
Ctg13_104	Trigger factor
Ctg13_105	ATP-dependent Clp endopeptidase proteolytic subunit ClpP
Ctg13_106	ATP-dependent Clp protease ATP-binding subunit ClpX

Cluster 5: Siderophore

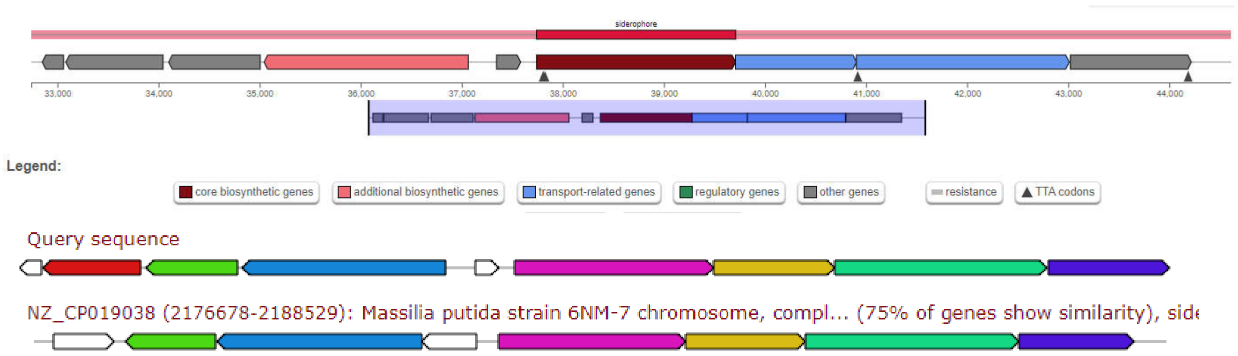

Contig No./locus tag	Identified protein name
Ctg30_29	No significant similarity
Ctg30_30	Hypothetical protein
Ctg30_31	tRNA glutamyl-Q(34) synthetase GluQRS
Ctg30_32	EAL domain-containing protein
Ctg30_33	No significant similarity
**Ctg30_34**	**IucA/IucC family siderophore biosynthesis protein**
Ctg30_35	MFS transporter
Ctg30_36	TonB-dependent siderophore receptor
Ctg30_37	PepSY domain-containing protein

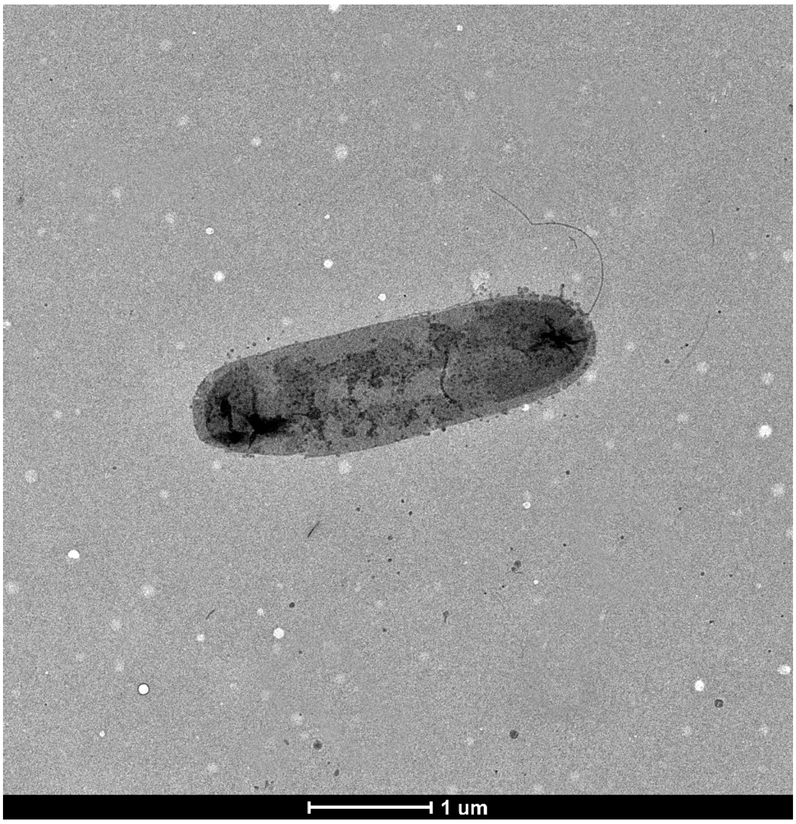

Supplementary Figure S5. Trasmission electron photomicrograph of strain TW-1^T grown on R2A agar at 15 °C for 5 days. Bar, 1 µm.

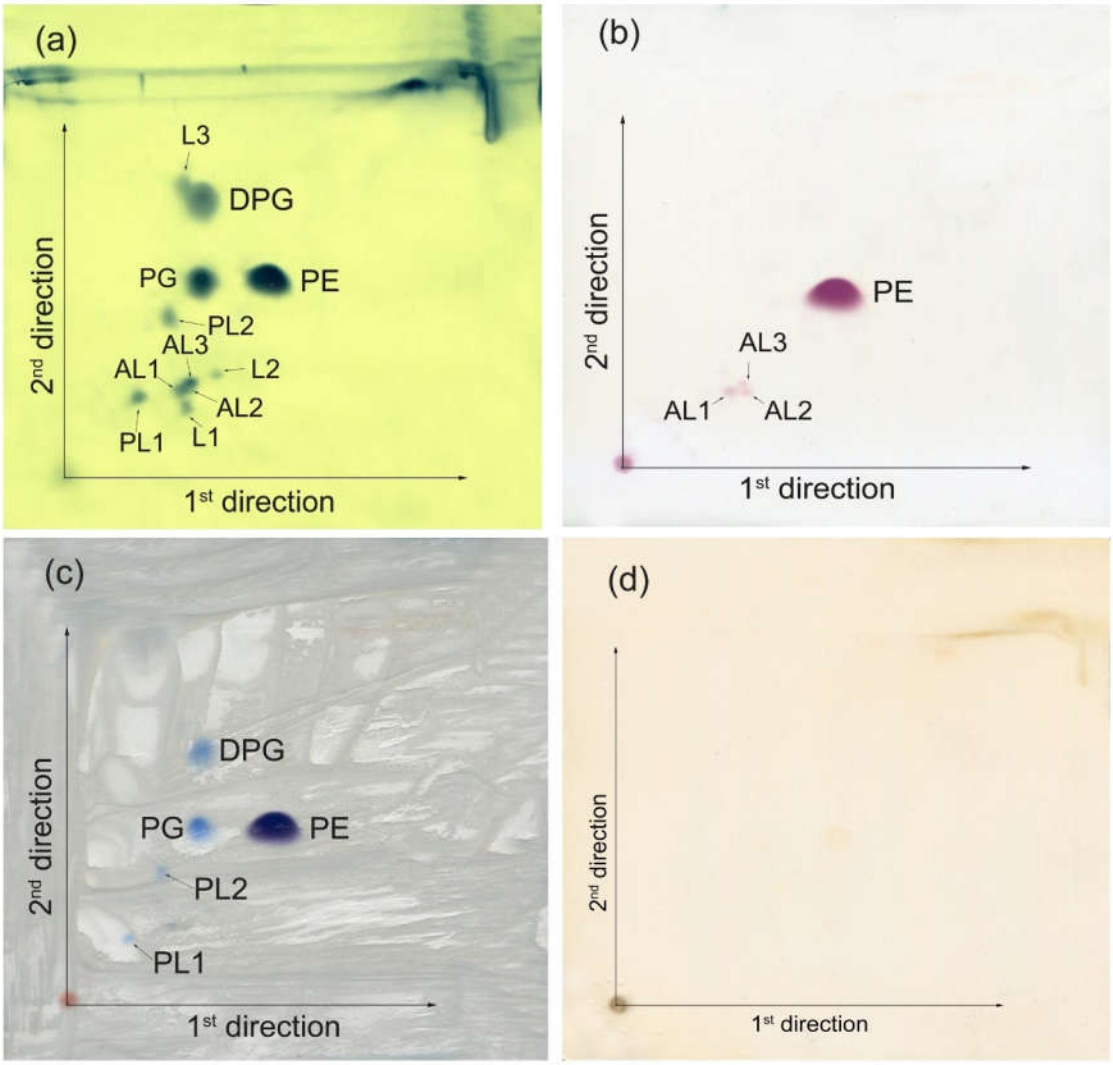

Supplementary Figure S6. Thin-layer chromatograms of the polar lipids from strain TW-1^{T}. The components were seen by staining with 5% molybdophosphoric acid in ethanol and heating them at 180 °C for 15 min (a); ninhydrin at 110 °C for 15 min (b); molybdenum at room temperature (c); and α-napthol-sulfuric acid at 110 °C for 15 min (d). Abbreviations: PE, phosphatidylethanolamine; DPG, diphosphatidylglycerol; PG, phosphatidylglycerol; AL1–AL3, unidentified aminolipids; PL1–PL2, unidentified phospholipids; L1–L3, unidentified polar lipids.

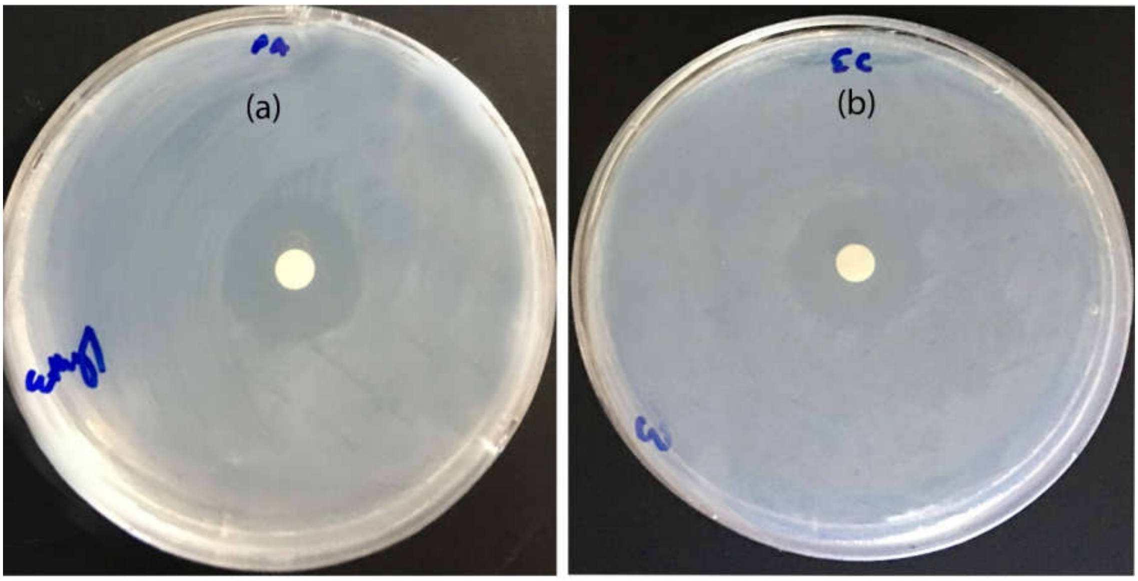

Supplementary Figure S7. Zone of inhibition shown by ethyl acetate extract of culture supernatant from strain TW-1^T against *Pseudomonas aeruginosa* KACC 10185 (a) and *Escherichia coli* KEMB 212-234 (b). For negative control 15 μL of methanol in 6 mm paper disc was used.
